# Toward an intergenerational model of metabolic and bariatric surgery: reframing the household as the unit of care

**DOI:** 10.1038/s41366-026-02131-1

**Published:** 2026-06-18

**Authors:** Alaina P. Vidmar, Kamran Samakar, Matthew J. Martin, John Magaña Morton

**Affiliations:** 1 Department of Pediatrics, Center for Endocrinology, Diabetes and Metabolism, Children’s Hospital Los Angeles and Keck School of Medicine of USC, Los Angeles, CA USA; 2 Division of Pediatric Surgery, Department of Surgery, Children’s Hospital Los Angeles and Keck School of Medicine of USC,, Los Angeles, CA USA; 3 Division of Upper Gastrointestinal and General Surgery, Department of Surgery, Keck Medical Center of University of Southern California, Los Angeles, CA USA; 4 Department of Surgery, Yale School of Medicine, New Haven, CT USA

**Keywords:** Obesity, Paediatrics, Weight management

## Abstract

Obesity is a chronic, relapsing disease that increasingly spans generations within households, yet contemporary metabolic and bariatric surgery (MBS) models remain oriented toward individuals or single-generation patients. A growing body of evidence demonstrates that obesity risk, behaviors, and treatment responses cluster within family systems, and that MBS produces measurable metabolic and behavioral effects among untreated spouses, partners, and children. Concurrently, decades of pediatric research confirm that caregiver engagement and integration is one of the strongest determinants of successful obesity treatment. Together, these observations support a paradigm shift toward an intergenerational, family-centered model of MBS, in which the household becomes the unit of care. This perspective synthesizes evidence supporting the biological, behavioral, and environmental interdependence of obesity within families and outlines a comprehensive framework for implementing household-level bariatric care. Core components include integrated pediatric–adult clinical infrastructure; combined multidisciplinary teams trained across the age spectrum; harmonized protocols for evaluation, education, and follow-up; coordinated scheduling and workflow alignment; and family-based behavioral strategies that promote shared goals, consistent routines, and mutual accountability. Operational and policy innovations, such as cross-departmental agreements, unified electronic health records, and coordinated billing can facilitate sustainable implementation. We further identify research priorities, including quantifying metabolic ripple effects among untreated family members, evaluating bundled household-level interventions, and developing validated metrics to assess changes in the home environment. Treating the household as the patient offers a promising strategy to enhance medical obesity treatment, surgical durability, improve adherence, and disrupt intergenerational transmission of obesity. As obesity increasingly presents as a family condition, an intergenerational MBS model may help realign treatment with the realities of lived experience and improve outcomes across generations.

## Introduction

Obesity is a chronic, relapsing, multifactorial disease that increasingly manifests within households and across generations [1]. Clinicians should approach obesity with the expectation that it frequently affects multiple members of a household, and that effective management requires alignment of treatment strategies across the family unit rather than focusing solely on the individual. More than 40% of adults and 20% of youth currently live with obesity [2], yet prevailing treatment models remain centered on the individual despite strong evidence that obesity risk, behaviors, and outcomes cluster within families [3]. Household systems shape dietary patterns, physical activity, emotional regulation, and treatment adherence throughout the lifespan, yet traditional metabolic and bariatric surgery (MBS) pathways rarely harness these family influences [3]. More than a decade of research demonstrates that when one family member undergoes MBS, spouses and cohabitating adults frequently experience collateral weight loss, improved diet quality, and greater physical activity, even without direct intervention [4–6]. In parallel, pediatric obesity literature consistently shows that caregiver behaviors, including food purchasing, meal structure, and modeling of physical activity, are among the strongest modifiable determinants of child outcomes [3–5]. These converging lines of evidence suggest that obesity is not an individual disease expressed within families, but a condition actively mediated by household environments [3, 5]. These findings argue for a conceptual shift toward an intergenerational, family-centered model of MBS, in which the household becomes the core unit of care [3, 4]. This framework synthesizes emerging evidence supporting an intergenerational paradigm, outlines future research, and operational priorities for advancing family-centered obesity treatment. Rather than positioning caregivers as facilitators of pediatric treatment, it explicitly integrates pediatric and adult metabolic and bariatric pathways into a unified clinical model.

## Rationale for an intergenerational paradigm

Obesity risk and treatment response are profoundly shaped by family systems. Family Systems Theory proposes that individual behaviors emerge from the shared norms, routines, and communication patterns within a household [5, 7]. Within this framework, the household “meal-preparer” represents a critical leverage point; interventions targeting this individual often lead to broader improvements in dietary quality and nutritional patterns across all family members. Epidemiologic data confirm that children with caregivers who have obesity face substantially greater lifetime risk [5], and that caregiver behaviors, such as food purchasing, meal structuring, and modeling of physical activity, are among the strongest modifiable determinants of pediatric obesity [5]. Intergenerational influences extend beyond behavioral pathways; offspring conceived following maternal metabolic bariatric surgery show both short-term improvements in perinatal outcomes as well as longer-term benefits such as a lower long-term risk of obesity and improved metabolic parameters compared with siblings conceived prior to surgery, indicating meaningful biological and environmental transmission effects [4, 5, 7, 8].

Beyond risk transmission, treatment itself exerts cross-member effects [4, 6, 8]. Studies in adults consistently demonstrate a “surgical halo effect” in which untreated spouses and cohabitants lose weight and adopt healthier behaviors after another family member receives MBS (Table 1) [6, 8]. Functional support, including shared meal preparation and aligned grocery purchasing, predicts better long-term weight loss in treated adults [6, 8, 9]. Adults who have undergone metabolic bariatric surgery also have an improved understanding of the role of surgery in the multi-modal treatment of obesity, and may increasingly seek and support medical and/or surgical treatment options for their children or other family members [4, 8, 10]. These findings underscore a core principle: the home environment is an active mediator of medical and surgical success [3]. Similarly, pediatric literature shows that family-based interventions outperform child-only models, with caregiver engagement improving adherence, retention, and behavioral change [5, 7]. Taken together, these data suggest that MBS has intergenerational metabolic ripple effects and that integrating treatment across family members may enhance outcomes for all.

**Table 1:** Key studies demonstrating household effects of bariatric surgery.

Authors	Design	Participants	Mean BMI	Weight loss	*p*-values/CIs	Behavioral outcomes
Hosseinimoghadam et al. [6]	Randomized clinical controlled trial; family-centered empowerment model (FCEM) in bariatric patients	30 bariatric patients, 15 to FCEM intervention, 15 to control	Not specified in extraction; typical bariatric patients (obese)	Significant difference in weight loss between the two groups pre- and post-intervention (magnitude not specified)	Self-management: *p* = 0.3 (NS); General adherence: *p* = 0.2 (NS); Total perceived social support: *p* = 0.015; Family support: *p* = 0.006; Friends support *p* = 0.037	Perceived social support improved significantly in intervention vs. control; Self-management behaviors and general adherence unchanged
Moradi et al. [10]	Mixed cohort (retrospective-prospective), all children aged equal or lower than 5 years old, born to mothers referred to single center obesity clinic	A total of 56 children with an age range of 59 (60–1) months entered the study; 28 born to mothers with a history of bariatric surgery, and 28 born to mothers with obesity but without bariatric surgery	BMI percentiles of youth born to mothers with history of surgery: 54% OW/OB; without history of surgery: 57% OW/OB	There was a partial correlation between maternal bariatric surgery and child height.	At birth, weight and BMI were lower in the group with maternal bariatric surgery than in the control group (*P* = 0.02 and *P* = 0.03, respectively).	Maternal weight loss was respectively related to lower birth weight and child height.
Tan et al. [8]	Retrospective cohort; bariatric surgery	359 adults who underwent bariatric surgery	42.6 ± 7.7 kg/m2	Measured as %TWL; marital satisfaction predicts sustained weight loss; specific values not provided, but model shows β = 0.92 (SE = 0.37), 95% CI (0.18, 1.65), p = 0.02	β for marital satisfaction × Time 0.92 (0.37); 95% CI: 0.18, 1.65; *p* = 0.02; other factors NS	Family support was not a significant predictor of T2DM remission; focus on marital satisfaction
Tashiro et al. [11]	Retrospective cohort; bariatric surgery	328 youth (14-18) who underwent sleeve gastrectomy	Operative BMI: Parent history of bariatric surgery: 50.3 (10.9) kg/m^2^; No parental history of surgery: 47.8 (8.47) kg/m^2^	There was no difference in postoperative absolute change in BMI or percent change in BMI at any time among patients with and without a parental history of bariatric surgery.	Youth with a history of parental bariatric surgery (*N* = 76) and those without (*N* = 184) showed similar BMI change trajectories postoperatively. Preoperative %ΔBMI was 1.36 (SD 16.5) vs. 2.26 (SD 12.8), *p* = 0.6. Postoperative reductions were −15.2% (SD 11.7) vs. −17.3% (SD 11.2), *p* = 0.2. At 3 months, −13.3% vs. −14.9%, *p* = 0.3; at 6 months, −17.9% vs. −21.2%, *p* = 0.166; and at 12 months, −21.0% vs. −24.5%, *p* = 0.2.	Parental surgery history did not significantly influence youth BMI reduction outcomes.
Van De Maele et al. [12]	Cross-sectional cohort; children of mothers with bariatric surgery	36 BS; 71 OW/OB; 36 NW (total 143 children)	N/A	Not applicable (children born after maternal surgery)	Prevalence differences: p = 0.004; Breakfast skipping: significant (*p* = 0.023) with higher BMI SDS; BIA differences: *p* < 0.001 for father education, etc.	Children of mothers who underwent bariatric surgery consumed less salty snacks (some differences reported; e.g., consumption sizes: 55.9% >25 g vs. 82.5% OW/OB and 82% NW) and more low-calorie sweetened beverages (*p* = 0.01) and less fruit juice (*p* = 0.01). Breakfast skipping correlates with higher BMI SDS; no other anthropometric differences.
Aarts et al. [9]	Prospective observational; spouses and children of bariatric patients	88 partners, 20 adult children ( > 18), 25 youth (12–17) at baseline; 83 partners, 47 youth at 1-year follow-up	N/A	Partners: 66.2% lost weight; 8.1% stable; 25.7% gained weight; BMI decreased significantly (*p* = 0.002), especially partners with overweight (p < 0.001); Children: no significant change	Partners: *p* = 0.002 overall; Overweight partners: *p* < 0.001; Eating behavior: no significant changes	No significant changes in eating behaviors among partners or children
Woodard et al. [4]	Prospective, longitudinal, cohort study	35 families of patients who underwent bariatric surgery at an academic medical center	Preoperative BMI of family members: 33 kg/m^2^ Preoperative BMI of patient: 48.7 kg/m^2^ Preoperative BMI of children: 26.3 kg/m^2^	Family member: Adult family members with obesity lost weight from 234 lb to 226 lb at 12 months (Δ = −8 lb), *P* = 0.01. Children: Trend toward lower BMI than expected for growth curve (expected 31.2 vs. observed 29.6), *P* = 0.07.	Family member weight loss: *P* = 0.01 Child BMI trend: *P* = 0.07 Family member activity increase: *P* = 0.005 Child activity increase: *P* = 0.04 Eating behavior improvements: Uncontrollable eating: *P* = 0.01 Emotional eating: *P* = 0.04 Alcohol consumption: *P* = 0.009	Physical Activity: Adults increased from 8 to 17 MET-hours/day; children from 13 to 22 MET-hours/day. Eating Habits: Adults reduced uncontrollable eating (35 → 28), emotional eating (36 → 28), and alcohol intake (11 drinks/month → 1 drink/month). Children: Showed BMI improvement trend and increased activity.

## Limitations of current models

Despite compelling evidence, current MBS pathways remain fragmented [3, 5]. Adult programs rarely integrate caregivers [7, 8]; pediatric obesity care depends on caregivers but is structurally disconnected from adult surgical programs [7]; and existing reimbursement models disincentivize joint visits and shared education. These siloes foster inconsistent messaging, undermine postoperative dietary adherence, and miss the opportunity to reshape household routines that strongly influence treatment durability [1, 5]. The rise of obesity medications further amplifies the need for synchronized household-level interventions, as misaligned home food environments may counteract medication-induced appetite changes in the treated individual [5, 7]. A family-centered model offers a timely mechanism to address these inconsistencies by aligning biology, behavior, and environment at the level where obesity is most consistently shaped, the home [3, 7].

## Treating the household as the patient

An intergenerational, family-centered MBS model reframes obesity treatment by positioning the household, not the individual, as the fundamental unit of care. Implementing such a model requires integrated pediatric–adult clinical infrastructure, including co-located or tightly partnered clinics, unified electronic medical record access, and multidisciplinary teams jointly trained across the age spectrum (Table 2) [4–6]. This approach requires clinicians to explicitly assess which members influence food procurement, preparation, and daily routines, and to direct interventions accordingly. Importantly, this model must be adaptable to non-traditional and alternative family structures, including shared custody arrangements, multihousehold caregiving, and variable caregiver engagement. Strategies such as telehealth-enabled participation, asynchronous education modules, and flexible scheduling allow inclusion of multiple caregivers across different environments. Harmonized protocols for evaluation, candidacy screening, nutrition and psychological assessment, preoperative preparation, and postoperative follow-up ensure consistency across generations.

**Table 2:** Core components and infrastructure for an intergenerational (family-centered) metabolic and bariatric surgery (MBS) Model.

Component	Operational strategy/description
Household assessment and family readiness	Assess household stress, social support, food insecurity, daily routines, caregiving responsibilities, transportation barriers, and multigenerational readiness for lifestyle and surgical commitments.
Integrated clinical setting	Co-locate pediatric and adult bariatric services, or create tightly affiliated clinics, to facilitate warm handoffs, reduce fragmentation, and support coordinated care. Includes unified EMR access to streamline communication across providers.
Combined multidisciplinary team	Joint pediatric and adult teams consisting of bariatric surgeons, obesity medicine clinicians, dietitians, psychologists, and dedicated social workers as essential team members trained across the age spectrum. Social workers play a central role in addressing social determinants, coordinating care across fragmented family systems, and maintaining functional household engagement.
Unified protocols and guidelines	Harmonize evaluation criteria, candidacy screening, nutritional and psychological assessments, preoperative preparation, and postoperative follow-up using evidence-based ASMBS, AAP, and AACE guidelines for both youth and adults.
Family-based education and support	Deliver standardized, shared pre- and postoperative education to participating family members, including nutrition, physical activity, behavioral strategies, and group support sessions. Group education may align with DSMT or MNT billing pathways.
Dyadic and family behavioral strategies	Implement shared household goals, family-level behavioral contracts, coordinated meal planning, structured routines, and reciprocal accountability to reinforce consistent habits across generations.
Coordinated scheduling and workflow alignment	Align evaluations, educational sessions, surgical dates, and postoperative visits for family members. Streamline workflows to maximize convenience, enhance adherence, and create shared treatment experiences.
Institutional partnerships and governance	Establish cross-department MOUs to enable shared resources, staffing, and collaborative care planning between pediatric and adult systems. Supports cross-subsidization and sustainability in programs historically reimbursed at different levels.
Billing and reimbursement strategies	Use separate but coordinated billing for pediatric and adult services; employ group visit codes for shared education; and explore family-based bundled payments or value-based care models that support multigenerational obesity treatment.
Data infrastructure and documentation	Maintain unified EMR access, shared documentation templates, and coordinated care plans to track interventions across family members and providers.
Household-level and longitudinal metrics	Track individual and family-level outcomes, including weight, metabolic measures, psychosocial functioning, household adherence, food environment changes, and quality of life. Incorporate long-term follow-up to assess generational impact.
Future research and evaluation	Evaluate comparative effectiveness of intergenerational vs. individual-based models, long-term weight and metabolic outcomes, psychosocial effects, and validated family-level metrics. Develop policy and reimbursement strategies to sustain family-centered care.

Central to this approach is a structured assessment of family readiness, addressing social support, stress, food insecurity, caregiving demands, and transportation barriers [8]. Special attention should be given to discordant caregiver motivation, with tailored engagement strategies to align goals across households when feasible. Shared educational pathways, such as joint nutrition classes, postoperative support groups, and behavioral skills training, provide consistent messaging and prepare families for the parallel lifestyle transitions required after surgery. Coordinated scheduling systems that align evaluation visits, education, surgery dates, and follow-up appointments reduce fragmentation, enhance adherence, and strengthen mutual accountability. Together, these components create a unified care pathway that reflects the biological, behavioral, and social reality that obesity is a shared family condition.

## Operational, financial, and research considerations for intergenerational metabolic and bariatric surgery

Delivering household-level bariatric care requires institutional alignment and innovative policy strategies. Several barriers must be proactively addressed to ensure feasibility and scalability. These include fragmented family structures, socioeconomic constraints, limited caregiver availability, and misaligned incentives within healthcare systems. To overcome these barriers, programs can implement flexible care delivery models, including evening/weekend clinics, telehealth visits, and hybrid education platforms that allow participation across multiple households. Transportation and access barriers may be mitigated through community partnerships and virtual care pathways. The inclusion of social workers as core, non-optional members of the multidisciplinary team is critical. Social workers play a central role in addressing social determinants of health, facilitating communication across caregivers, supporting family cohesion, and navigating complex psychosocial dynamics that directly influence treatment adherence and durability.

Cross-department memoranda of understanding, joint staffing models, and shared documentation templates support seamless collaboration between pediatric and adult health systems and offer opportunities for cross-subsidization in historically underfunded pediatric programs. Reimbursement remains a key barrier; however, coordinated billing for individual family members, use of diabetes self-management training (DSMT) or medical nutrition therapy (MNT) codes for group education, and emerging value-based or bundled family payment models may enable sustainable implementation [1]. A robust data infrastructure is essential, including shared electronic medical records access and longitudinal tracking of both individual and household-level outcomes, such as dietary environment shifts, psychosocial functioning, and metabolic changes among treated and untreated family members.

## Future research priorities

To establish a robust evidence base for intergenerational MBS, future studies should quantify metabolic and behavioral changes among untreated household members, test bundled household-level interventions combining surgery, pharmacotherapy, and behavioral treatment, and identify which family dynamics most strongly predict long-term outcomes [5, 8]. Large-scale epidemiologic analyses should examine the impact of metabolic bariatric surgery in parents on obesity health literacy, active management of obesity in their children or other family members, and patterns of seeking referral for medical and surgical obesity management programs. Research should also explore biological mechanisms underlying intergenerational effects, including microbiome and epigenetic pathways, and examine cost-effectiveness compared with standard individual care. Ultimately, measuring success at the household, not individual, level may reveal benefits currently overlooked by traditional models.

## Conclusion

The intergenerational model of metabolic and bariatric surgery represents a paradigm shift in obesity care. By integrating adult and pediatric programs, synchronizing behavioral pathways, and explicitly engaging families, while accounting for diverse family structures and addressing social and logistical barriers through multidisciplinary support, this approach leverages shared motivation, aligned environments, and mutual support to enhance surgical durability. As obesity increasingly spans generations within households, treating the family as the patient, not the individual, offers a promising path toward breaking intergenerational cycles of disease and improving outcomes for future generations.

## Data Availability

The datasets from this study will be available from the corresponding author on written request.
